# Oral Osteomicrobiology: The Role of Oral Microbiota in Alveolar Bone Homeostasis

**DOI:** 10.3389/fcimb.2021.751503

**Published:** 2021-11-17

**Authors:** Xingqun Cheng, Xuedong Zhou, Chengcheng Liu, Xin Xu

**Affiliations:** State Key Laboratory of Oral Diseases, National Clinical Research Center for Oral Diseases, West China Hospital of Stomatology, Sichuan University, Chengdu, China

**Keywords:** oral microbiota, alveolar bone, osteomicrobiology, osteoimmunology, RANKL signaling, Notch signaling, Wnt signaling, synthetic microbial community

## Abstract

Osteomicrobiology is a new research field in which the aim is to explore the role of microbiota in bone homeostasis. The alveolar bone is that part of the maxilla and mandible that supports the teeth. It is now evident that naturally occurring alveolar bone loss is considerably stunted in germ-free mice compared with specific-pathogen-free mice. Recently, the roles of oral microbiota in modulating host defense systems and alveolar bone homeostasis have attracted increasing attention. Moreover, the mechanistic understanding of oral microbiota in mediating alveolar bone remodeling processes is undergoing rapid progress due to the advancement in technology. In this review, to provide insight into the role of oral microbiota in alveolar bone homeostasis, we introduced the term “oral osteomicrobiology.” We discussed regulation of alveolar bone development and bone loss by oral microbiota under physiological and pathological conditions. We also focused on the signaling pathways involved in oral osteomicrobiology and discussed the bridging role of osteoimmunity and influencing factors in this process. Finally, the critical techniques for osteomicrobiological investigations were introduced.

## 1 Introduction

Humans are inhabited by a diverse milieu of microorganisms, referred to as the commensal microbiota. They mostly reside in five body regions: the gut, oral cavity, skin, nose, and vagina, and are essential for human development, nutrition, and immune status. Accumulating evidence has indicated a close connection between the commensal microbiota and bone health. In 2012, Sjögren et al. demonstrated an increased bone mass in germ-free (GF) mice compared to controls raised in conventional conditions. This phenotype was reversed by colonization with gut flora from conventionally raised mice, providing evidence that the results were not due to innate abnormalities of the GF mice (Sjögren et al., 2012). That was the first report to suggest that the gut microbiota is a critical regulator of bone mass. Two years later, Ohlsson and Sjögren introduced a new term, “osteomicrobiology”, to refer to the study of the role of microbiota in health and disease, and the mechanisms by which microbiota regulate post-natal skeletal maturation, bone aging, and pathological bone loss (Ohlsson and Sjögren, 2015).

Recently, numerous links have been suggested between the gut microbiota and bone remodeling (Ohlsson and Sjögren, 2018; Yan et al., 2018). In general, the effect of the gut microbiota on bone depends on various factors, such as the composition of the microbiome, human diet, and age (Yan et al., 2018). However, the mechanisms by which the gut microbiota participate in bone regulation require further investigation. The oral cavity houses the second largest and second-most diverse microbiota after the gut in the body, with over 700 species of bacteria, fungi, viruses, archaea, and protozoa currently known (Paster et al., 2006). The alveolar bone is that part of the maxilla and mandible that supports the teeth, and the association of the oral microbiota with alveolar bone homeostasis has also received considerable attention (Costalonga and Herzberg, 2014; Hathaway-Schrader and Novince, 2021). In 1969, Brown et al. first reported that alveolar bone loss is statistically significantly stunted in GF mice compared with specific-pathogen-free (SPF) mice (Brown et al., 1969). More recently, Hajishengallis et al. validated those results (Hajishengallis et al., 2011). Moreover, an interplay between the oral microbiota and immune and bone cells was demonstrated by Horton et al. in 1972. Specifically, human peripheral blood leukocytes stimulated by dental plaque derived from patients with periodontitis produced osteoclast-activating factors (calcium-45) and increased the number of active osteoclasts (Horton et al., 1972). Collectively, these studies indicate that there is a complex, reciprocal relationship between the oral microbiota and alveolar bone homeostasis. Depending on the conditions, oral microbiota may have either a protective or a pathological effect on alveolar bone. However, the available data suggest that such interaction is limited, and the mechanism underlying alveolar bone regulation by the oral microbiota remains to be elucidated. Thus, we propose the term “oral osteomicrobiology” to denote the rapidly emerging field of study of the role of oral microbes in alveolar bone health and disease, aiming to bridge the gaps in the interplay between oral microbiology, immunology, and the alveolar bone.

Patients with severe periodontitis are estimated to swallow 10^12^–10^13^ bacteria in their saliva daily (Sender et al., 2016). Swallowed indigenous oral bacteria can change the composition of the gut microbiota and induce gut dysbiosis (Kitamoto et al., 2020; Kobayashi et al., 2020). Moreover, intestinal microorganisms can indirectly affect the structure of the oral microbiome. Inflammatory bowel disease is an inflammatory response caused by intestinal flora disorders. Inflammatory bowel disease is often accompanied by changes in the composition of the salivary microbiota and corresponding oral symptoms, suggesting that the intestinal microbiota in the pathological state may directly or indirectly affect the composition of the oral microbiome (Said et al., 2014). Probiotics can also alter the composition and/or metabolic activity of gut microbiota, which can result in modulatory effects on the host immune response as well as oral microbiota (Abboud and Papandreou, 2019; Mishra et al., 2020). Therefore, oral and gut osteomicrobiota seemingly interact with each other. For example, mice with gut dysbiosis induced by orally administered *Porphyromonas gingivalis* have an increased immune response, worse arthritis, and substantially lower bone mineral density than do controls (Arimatsu et al., 2014; Sato et al., 2017). Trinitrobenzene sulphonic acid and dextran sodium sulphate treatment in mice elicited gut dysbiosis and caused alveolar bone loss in both maxillae and mandibles, worsening over time (Oz and Ebersole, 2010). Berberine ameliorates periodontal bone loss in rats by regulating the gut microbiota (Jia et al., 2019). Although there are similarities in the mechanisms involved in alveolar bone loss mediated by the oral and gut microbiota, there are also unique characteristics. Both the oral and gut microbiota regulate bone homeostasis by inducing the host immune response and sustained changes in receptor activator of nuclear factor kappa B (NF-κB) ligand (RANKL)-mediated osteoclastogenesis (Hsu and Pacifici, 2018). The gut microbiota can alter the production of insulin-like growth factor 1, and regulate nutrient absorption and metabolism, affecting the hormone production critical for bone health such as sex steroids, vitamin D, and serotonin (Markle et al., 2013; Hsu and Pacifici, 2018). The oral microbiota causes alveolar bone resorption when the ecological equilibrium is disturbed. During the pathological process, virulence factors of pathobionts play important roles (Costalonga and Herzberg, 2014). However, the direct linkages and differences between oral and gut osteomicrobiology have not been elucidated.

In the present article, to contribute to the understanding of oral osteomicrobiology, we review the roles of the oral microbiota in alveolar bone formation and loss, discuss the role of osteo-immunomodulatory effects as a bridge between the oral microbiota and the alveolar bone, and inspect the mechanisms by which the oral microbiota modulate alveolar bone. We focused on RANKL, Notch, and Wingless-integrated (Wnt) signaling, as well as the nucleotide oligomerization domain-like receptor family pyrin domain-containing 3 (NLRP3) inflammasome. We also summarize the factors that influence the interaction between the oral microbiota and alveolar bone loss, as well as techniques that are critical for oral osteomicrobiology research.

## 2 Oral Microbiota in Alveolar Bone Formation and Bone Loss

### 2.1 Oral Microbiota in Post-Natal Jawbone Development

The gut commensal microbiota have been demonstrated to affect bone remodeling. For instance, GF mice have a general growth defect reflected by a slower gain in body weight as well as decreased longitudinal and radial bone growth compared to conventionally raised mice. This is due to growth hormone resistance and a reduced concentration of insulin-like growth factor 1 concentrations, both associated with the gut microbiota; their phenotype can be normalized by treatment with a specific *Lactobacillus plantarum* strain (Schwarzer et al., 2016). As for the oral microbiota, several lines of evidence have suggested that they participate in regulating post-natal jawbone development. SPF mice reportedly have a larger body size with a lower alveolar bone mineral density and alveolar bone volume fraction compared with GF mice (Uchida et al., 2018). Further analysis suggested that the oral commensal microbiota prevent excessive mineralization by enhancing the expression of osteocalcin, an inhibitor of bone mineralization, in osteoblasts, and directs the activity of osteoblasts and osteoclasts by regulating specific transcription factors (Uchida et al., 2018). For example, the expression of *androgen receptor* and *alkaline phosphatase* was activated in SPF mice, which increased long bone growth and size, and enhanced differentiation of osteoblasts, respectively (Huang et al., 2007; Manolagas et al., 2013). Existing data indicate that the commensal microbiota is responsible for both anabolic and catabolic activities in alveolar bone formation and physiological skeletal growth (Sjögren et al., 2012; Novince et al., 2017; Irie et al., 2018). Further investigations are required to clarify the regulation of post-natal jawbone development by the oral microbiota.

### 2.2 Oral Microbiota in Physiological Alveolar Bone Loss

The alveolar bone “lives and dies” with the teeth, as it forms during teeth development and eruption but is resorbed after tooth loss. In the physiological state, the alveolar bone is renewed through a succession of apposition-resorption cycles, with osteoclasts responsible for tissue resorption and osteoblasts for matrix deposition (Preshaw et al., 2007). The balance between those two opposite functions results in a dynamic equilibrium of constantly remodeled healthy bone. Disturbance of this delicate balance leads to excess bone loss (Harris and Heaney, 1969).

Aging is a process of physiological involution. Although alveolar bone loss is not a natural consequence of aging, both clinical and animal studies have indicated a positive correlation between alveolar bone loss and aging in physiological conditions. For example, Hajishengallis et al. revealed that aged, healthy GF mice (18-month-old) showed increased alveolar bone loss and concentrations of inflammatory mediators compared with young GF mice (5-week-old) (Hajishengallis et al., 2011). They also demonstrated that the commensal microbiota was necessary for and directly contributed to the non-pathological bone loss observed in their model. In agreement, Liang et al. reported that old mice displayed a statistically significant increase in alveolar bone destruction, accompanied by an elevated expression of proinflammatory cytokines, in comparison with young mice, suggesting that alveolar bone is resorbed to a greater extent with age (Liang et al., 2010). A more recent study of the effects of aging on periodontal tissues revealed that SPF but not GF mice exhibited an age-related increase in alveolar bone loss (Irie et al., 2018). In healthy humans, a modest but not critical loss of periodontal support has been discovered with age (Huttner et al., 2009). This “natural” bone loss is associated with an increase in periodontal cell response to the oral microbiota, alterations in differentiation and proliferation of the osteoblasts and osteoclasts, and endocrine alterations (Nishimura et al., 1997; Abiko et al., 1998; Okamura et al., 1999; Huttner et al., 2009). The specific mechanisms of physiological alveolar bone loss remain unknown. Gut microbiota have an anti-anabolic effect by inhibiting osteoblastogenesis and a pro-catabolic effect by stimulating osteoclastogenesis, ultimately driving bone loss (Novince et al., 2017); oral commensal microbiota may have the same effects on physiological alveolar bone loss. Natural bone loss seems to be a manifestation of the homeostatic relationship between the host and its oral microbial community. Moreover, further study is required to determine whether the oral commensal microbiota directly affects physiological alveolar bone loss, and which features of the oral microbiome predispose individuals to bone loss. The techniques needed to study these issues will be introduced in section 5.

### 2.3 Oral Microbiota as Regulator of Pathological Alveolar Bone Loss

The dysbiosis of the oral microbiota results in an increase in pathogenic microorganisms or in the pathogenicity of the microbiota. The oral microbiota has a catabolic effect, impacting osteoclast-osteoblast-mediated alveolar bone remodeling, leading to pathological bone loss. Most cases of pathological alveolar bone loss are associated with oral infectious diseases (e.g., periodontitis, apical periodontitis, and peri-implantitis) driven by the oral microbiota. The results of relevant studies have been summarized in **Table 1**.

**Table 1:** Summary of the dysbiosis of oral microbiota associated with pathological alveolar bone loss.

Diseases associated with alveolar bone loss	Principle findings of pathogens associated with alveolar bone loss	Animal model or clinical study	References
Periodontitis	*P. gingivalis* colonization facilitated a change in quantity and composition of the commensal oral microbiota.	Mouse models	Hajishengallis et al., 2011; Sato et al., 2018; Darveau et al., 2012
	Oral microbiota in *P. gingivalis*-treated mice exhibited lower concentrations and an imbalance, with decreased proportions of taxa associated with good oral health.	Mouse model	Boyer et al., 2020
	Concentrations of antibodies against *P. gingivalis* W83 and/or 381, *E. corrodens*, and *P. gingivalis* 33277 were positively correlated with alveolar bone loss, while the number of enteric bacteria and concentrations of antibodies against *F. nucleatum* and *P. intermedia* were negatively correlation with alveolar bone height.	Clinical study	Wheeler et al., 1994
	*P. gingivalis* and *T. denticola* concentrations were associated with the degree of alveolar bone loss.	Clinical study	Pradhan-Palikhe et al., 2013
	Healthy participants had higher concentrations of *Streptococcus* and *Actinomyces* sp., while participants with bone loss had higher concentrations of *A. actinomycetemcomitans*, *S. parasanguinis*, *F. alocis*, *P. micra*, and *Peptostreptococcus* sp. human oral taxon 113.	Clinical study	Fine et al., 2013
Apical periodontitis	A spectrum of 300 species colonizing the healthy human mouth have been consistently isolated from infected root canals of teeth with periapical destruction.	Clinical study	Nair, 1997
	The prominent isolates in apical periodontitis included *Enterococcus*, *Eubacterium*, *Fusobacterium*, *Campylobacter*, *Porphyromonas*, *Prevotella*, *Peptostreptococcus*, *Propionibacterium*, and *Streptococcus* strains.	Clinical studies	Farber and Seltzer, 1988; Sundqvist et al., 1989
	The root canal microbiome is dominated by aerobic and facultative anaerobic bacteria during the early course of pulpal infection; thereafter, obligate anaerobes become more abundant.	Clinical studies	Tani-Ishii et al., 1994; Stashenko et al., 1994
	Proper endodontic treatment resulted in substantial or complete radiographic regression of apical periodontitis, whereas persisting symptoms were associated with either incomplete closure of the root canal chamber or improper disinfection.	Clinical studies	Orstavik, 1996; Sundqvist, 1994
Peri-implantitis	The most commonly reported bacteria associated with peri-implantitis were obligate anaerobe Gram-negative bacteria, asaccharolytic anaerobic Gram-positive rods, and other Gram-positive species.	Clinical study	Kensara et al., 2021
	The peri-implantitis microbiome is commensal-microbe-depleted and pathogen-enriched, with increased concentrations of *Porphyromonas* and *Treponema* sp.	Clinical study	Sanz-Martin et al., 2017
	The core peri-implantitis-related species were *Fusobacterium, Parvimonas*, and *Campylobacter* sp., as well as organisms often associated with periodontitis (*T. denticola*, *P. gingivalis*, *F. alocis*, *F. fastidiosum*, and *T. maltophilum*).	Clinical study	Sanz-Martin et al., 2017
	*S. moorei* and *P. denticola* were core taxa specific to peri-implantitis.	Clinical study	Komatsu et al., 2020
	Implants caused bone loss at remote periodontal sites due to microbial dysbiosis induced by the implants.	Clinical study	Heyman et al., 2020
	*Firmicutes* decreased and *Bacteroides* increased in the peri-implantitis group at the phylum level, and *Peptostreptococcus* decreased and *Porphyromonas* increased at the genus level.	Canine model	Qiao et al., 2020

P. gingivalis, Porphyromonas gingivalis; E. corrodens, Eikenella corrodens; F. nucleatum, Fusobacterium nucleatum; P. intermedia, Prevotella intermedia; T. denticola, Treponema denticola; A. actinomycetemcomitans, Aggregatibacter actinomycetemcomitans; S. parasanguinis, Streptococcus parasanguinis; F. alocis, Filifactor alocis; P. micra, Parvimonas micra; F. fastidiosum, Fretibacterium fastidiosum; T. maltophilum, Treponema maltophilum; S. moorei, Solobacterium moorei; P. denticola, Prevotella denticola.

#### 2.3.1 Periodontitis

Periodontitis is a chronic inflammatory disease affecting tooth-supporting tissues. It is initiated by microbial communities but requires disruption of the normal host immune-inflammatory state (Curtis et al., 2020). Moreover, periodontitis is a dysbiosis disease, reliant upon an entirely dysfunctional oral microbiome, not a conventional infectious disease caused by select periodontal pathogens (Hajishengallis et al., 2011; Hajishengallis and Lamont, 2021). Polymicrobial communities induced a dysregulated host response and resulted in periodontal tissue destruction (Lamont et al., 2018). GF mice administered with *P. gingivalis* did not develop any detectable pathogenic changes, while *P. gingivalis* induced bone loss and substantial changes in the oral commensal microbial community in SPF mice, indicating that an oral microbial shift is critical for *P. gingivalis*-induced alveolar bone loss (Hajishengallis et al., 2011; Sato et al., 2018). Consistent with that result, Darveau et al. discovered that *P. gingivalis* could modulate the complement function, facilitating a marked change in both the quantity and composition of the commensal oral microbiota, ultimately contributing to pathological bone loss in mice (Darveau et al., 2012). Interestingly, the ability of *P. gingivalis* to cause oral microbiota-mediated alveolar bone loss is strain-dependent. For instance, *P. gingivalis* W83 can reportedly initiate periodontitis, while *P. gingivalis* TDC60-treated mice experience only moderate lesions. *P. gingivalis* W83-treated mice reportedly exhibit a substantial reduction, imbalance, and shift in the proportions of microbial taxa compared to healthy mice (Boyer et al., 2020).

In the disrupted periodontal microenvironment, Gram-negative bacteria dominate. This dysbiosis induces inflammation and a loss of the periodontal tissues. A cross-sectional periodontal study indicated that the concentrations of antibodies to *P. gingivalis* W83 and/or 381, *Eikenella corrodens*, and *P. gingivalis* 33277 were all positively correlated with alveolar bone loss, while the number of enteric bacteria and concentrations of antibodies to *Fusobacterium nucleatum* and *Prevotella intermedia* were all negatively correlated with alveolar bone height (Wheeler et al., 1994). The concentrations of microbial species considered etiologically related to periodontitis, such as *P. gingivalis* and *Treponema denticola*, were statistically significantly associated with the degree of alveolar bone loss (Pradhan-Palikhe et al., 2013). In a longitudinal study, it was demonstrated that a test for *Aggregatibacter actinomycetemcomitans* was positive in 91.7% of participants presenting with vertical periodontal bone loss, highlighting the destructive pathological impact of that microorganism on the tooth-alveolar bone complex (Fine et al., 2013). Higher concentrations of *Streptococcus* and *Actinomyces* species were discovered in *A. actinomycetemcomitans*-positive participants who remained healthy, while higher concentrations of *A. actinomycetemcomitans*, *Filifactor alocis*, *Parvimonas micra*, and *Peptostreptococcus* sp. human oral taxon 113 were discovered in those with bone loss (Fine et al., 2013). At vulnerable sites, *A. actinomycetemcomitans*, *Streptococcus parasanguinis*, and *F. alocis* concentrations were elevated prior to bone loss. Taken together, data from that study reinforced the importance of *A. actinomycetemcomitans* in localized aggressive periodontitis and indicated a potential synergistic partnership of that microorganism with *F. alocis* and *S. parasanguinis* in non-junctophilin-2-related disease, as that consortium was strongly associated with alveolar bone loss (Fine et al., 2013). Fascinatingly, some human skulls, more than one thousand years of age, had pathogenic alveolar bone lesions in the tooth areas, characteristic of periodontitis (Philips et al., 2020). Microbiome analysis derived from the periodontitis sites indicated that the same pathogenic species were responsible for the development of periodontitis in those samples as are today (Philips et al., 2020). Taken together, there is strong evidence that the oral microbiota is closely associated with periodontitis-related alveolar bone loss. In particular, the shift of the oral flora to a predominance of gram-negative anaerobic bacteria plays a pivotal role in this process.

#### 2.3.2 Apical Periodontitis

Apical periodontitis is a prevalent infectious and inflammatory disorder that involves inflammation of periapical tissues and bone resorption surrounding the root apex (Wei et al., 2021). Ample clinical and experimental evidence indicates that apical periodontitis is initiated primarily by the mixed microflora of infected root canals (Márton and Kiss, 2000). A spectrum of 300 species colonizing the healthy human mouth have been consistently isolated from infected root canals of teeth with periapical destruction (Nair, 1997). The prominent isolates include *Enterococcus*, *Eubacterium*, *Fusobacterium*, *Campylobacter*, *Porphyromonas*, *Prevotella*, *Peptostreptococcus*, *Propionibacterium*, and *Streptococcus* strains (Farber and Seltzer, 1988; Sundqvist et al., 1989). The root canal microbiome is mainly dominated by aerobic and facultative anaerobic bacteria during the early course of pulpal infection, with obligate anaerobes increasing as a result of local consumption of oxygen (Stashenko et al., 1994; Tani-Ishii et al., 1994). Accumulating clinical follow-up studies have disclosed that proper endodontic treatment resulted in substantial or complete radiographic regression in 85% to 90% of apical periodontitis cases, whereas persisting symptoms were associated most frequently either with incomplete closure of the root canal chamber or improper disinfection, indicating the pathogenic role of the mixed bacterial flora of the pulp chamber in periapical infection (Sundqvist, 1994; Orstavik, 1996).

#### 2.3.3 Peri-implantitis

Peri-Implantitis is an infection of the tissue around an implant, resulting in the loss of supporting bone. A history of periodontitis, dental plaque, poor oral hygiene, smoking, diabetes, and alcohol consumption are risk factors for peri-implantitis (Nguyen-Hieu et al., 2012). Microbial involvement is one of the most important proposed etiological factors for bone loss around an implant (Bousdras et al., 2006). Mechanical treatment combined with antiseptics or antibiotics reportedly yields clinical attachment and bone reconstruction (Bousdras et al., 2006).

Microbial diversity and richness vary during peri-implantitis. The microbes most associated with peri-implantitis are obligate anaerobe Gram-negative bacteria, asaccharolytic anaerobic Gram-positive rods, and other Gram-positive species (Kensara et al., 2021). The peri-implantitis microbiome is commensal-depleted and pathogen-enriched, with an abundance of *Porphyromonas* and *Treponema* (Sanz-Martin et al., 2017) sp. The core peri-implantitis-related microbes were *Fusobacterium, Parvimonas*, and *Campylobacter* sp., as well as microbes often associated with periodontitis (*T. denticola*, *P. gingivalis*, *F. alocis*, *Fretibacterium fastidiosum*, and *Treponema maltophilum*) (Sanz-Martin et al., 2017). Komatsu et al. also deemed *Solobacterium moorei* and *Prevotella denticola* core taxa specific to peri-implantitis (Komatsu et al., 2020).

The immune response is triggered by the dysbiosis of the oral microbiota. The most frequently reported pro-inflammatory mediators associated with peri-implantitis are interleukin (IL)-1β, IL-6, IL-17, and tumor necrosis factor‐α (TNF-α). Osteolytic mediators such as receptor of NF-κB, RANKL, and Wnt5a, as well as proteinases such as matrix metalloproteinase-2, matrix metalloproteinase-9, and cathepsin-K are also reportedly upregulated in peri-implantitis sites compared to controls (Kensara et al., 2021). It is worth noting that implants have an impact on remote periodontal sites, as elevated inflammation and accelerated bone loss have been detected in intact, distant teeth (Heyman et al., 2020). That impact was due to microbial dysbiosis induced by the implants, since antibiotic treatment was demonstrated to prevent periapical bone loss. However, antibiotic treatment does not prevent the loss of implant-supporting bone, highlighting the distinct mechanisms mediating bone loss at each site (Heyman et al., 2020).

In experimental studies, placement of ligatures together with plaque formation causes resorption of supporting tissues and considerable inflammatory cell infiltration around implants and teeth (Berglundh et al., 2011). Using a canine peri-implantitis model, researchers observed that *Firmicutes* decreased and *Bacteroides* increased over time at the phylum level, and *Peptostreptococcus* decreased and *Porphyromonas* increased at the genus level (Qiao et al., 2020). They also identified several potential keystone species during peri-implantitis development using species-level and co-occurrence network analyses (Qiao et al., 2020). In summary, peri-implantitis is associated with a complex and distinct microbial community that includes bacteria, archaea, fungi, and viruses (Belibasakis and Manoil, 2021). The ecosystem shift from healthy to diseased includes an increase in microbial diversity and a gradual depletion of commensal microbes, along with an enrichment of classical and emerging periodontal pathogens. This change in the microbiota could provoke an inflammatory response and osteolytic activity, contributing to the physiopathology of peri-implantitis.

## 3 Osteomicrobial Mechanisms of Alveolar Bone Loss

**Figure 1:**
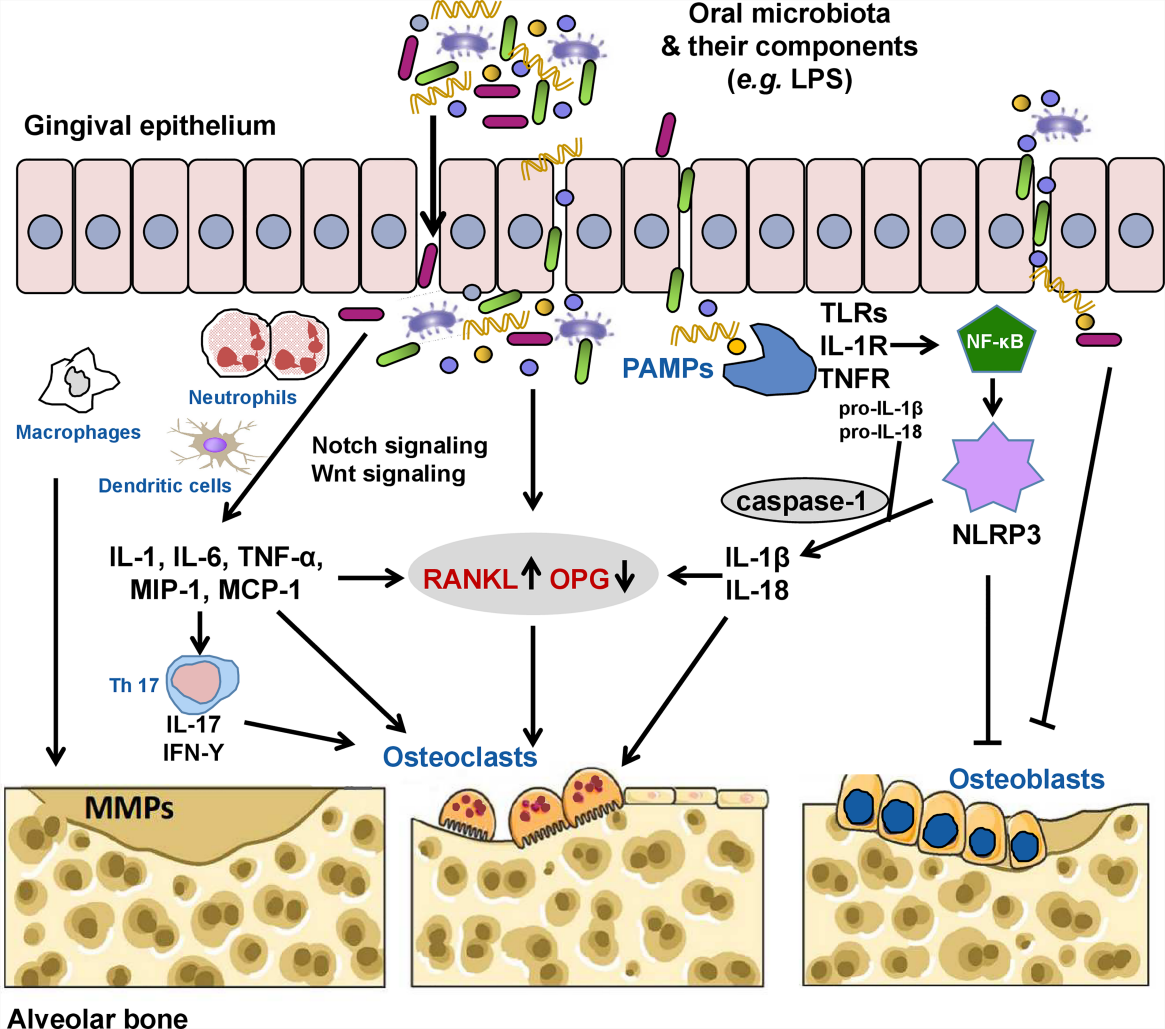
The oral microbiota and its components can invade the gingival epithelium through the production of proteases, thus activating receptor activator of nuclear factor kappa B (NF-κB) ligand (RANKL) signaling directly or indirectly by inducing the secretion of inflammatory cytokines (interleukin [IL]-1, IL-6, tumor necrosis factor [TNF]-α, macrophage inflammatory protein [MIP]-1, and monocyte chemoattractant protein [MCP-1]) by neutrophils, macrophages, and dendritic cells, increasing the RANKL/osteoprotegerin (OPG) ratio and contributing to alveolar bone loss by inducing osteoclast formation. Pathogenic T_H_17 cells stimulated by bacterial invasion evokes periodontal immune responses against these microorganisms or their metabolites while also inducing bone damage. Some pathogens (*e.g.*, *Porphyromonas gingivalis*) and their lipopolysaccharides (LPSs) can also directly induce the activation of matrix metalloproteinases (MMPs), which mediate the degradation of the extracellular matrix. Oral pathogen-associated molecular patterns (PAMPs) such as LPS, lipoteichoic acid, and double-stranded RNA can activate the innate immune system through pattern recognition receptors, including toll-like receptors (TLRs), IL-1 receptor (IL-1R), and TNF receptor (TNFR), causing the release of NF-κB into the nucleus to initiate the expression of the nucleotide oligomerization domain-like receptor family pyrin domain-containing 3 (NLRP3) inflammasome. Activated NLRP3 cleaves pro-caspase-1 into caspase-1. Caspase-1 promotes the maturation and release of pro-IL-1β and pro-IL-18 to induce secretion of RANKL and activate osteoclasts. NLRP3 and activated caspase-1 can also promote osteoblast apoptosis. In addition, the oral microbiota and/or microbial virulence factors can inhibit the differentiation and proliferation while promoting the apoptosis of osteoblasts *via* various mechanisms.

Pathological alveolar bone loss is net bone loss caused by increased osteoclastogenesis-mediated bone resorption and decreased osteoblastogenesis-mediated bone formation, a process that is mediated dynamically by both osteoclasts and osteoblasts. Under pathological conditions, oral pathogenic microbes or microbial dysbiosis induce catabolic disruption of osteoclast-osteoblast-mediated bone remodeling, which leads to alveolar bone loss. According to clinical, animal, and *in vitro* studies, microbial virulence factors and toxic derivatives could interfere with humoral or cellular anti-bacterial defense mechanisms, eliciting alveolar bone resorption (Márton and Kiss, 2000; Wei et al., 2021). As summarized in **Table 2**, the most typical such factor is lipopolysaccharide (LPS). It has been reported that 10^-3^ g/L LPS can directly stimulate bone loss, while a tiny concentration of LPS (10^-9^ g/L) can indirectly promote bone loss by activating the production of bone resorptive cytokines and prostaglandins (Beuscher et al., 1987; Paula-Silva et al., 2020). Interestingly, the indirect involvement of endotoxins in the process of alveolar bone loss is a million times more likely than a direct pathogenic role for this bacterial cell wall component (Beuscher et al., 1987; Tatakis et al., 1988). In particular, LPS could inhibit the differentiation and proliferation while promoting the apoptosis of osteoblasts *via* the following mechanisms: (1) inhibiting the expression of bone differentiation markers in osteoblast cells, including alkaline phosphatase, bone sialoprotein, and osteocalcin (Tachikake-Kuramoto et al., 2014); (2) substantially stunting synthesis of DNA and collagen (Wilson et al., 1988; Meghji et al., 1992); (3) elevating pro-inflammatory cytokine production of osteoblasts (Albus et al., 2016); and (4) inducing production of nitric oxide (Sosroseno et al., 2009). Moreover, a high concentration of *P. gingivalis* LPS could also reduce mesenchymal stem cell proliferation and osteogenic differentiation, and inhibit activated T cells (Tang et al., 2015). In addition, the capsular-like polysaccharide antigen from serotype c of *A. actinomycetemcomitans* inhibited osteoblast cell line proliferation through a pro-apoptotic mechanism (Yamamoto et al., 1999). It is more complex to determine how such factors and metabolites cause alveolar bone loss by regulating host signal transduction. Based on current evidence, RANKL, Notch, and Wnt signaling, as well as the NLRP3 inflammasome are major pathways involved in alveolar bone loss mediated by the oral microbiota (**Figure 1**). Osteoimmunity is the bridge that spans the gap between the microbiota and the bone.

**Table 2:** Summary of microbial virulence factors involved in alveolar bone loss.

Microbial virulence factors	Principle findings	References
LPS	10^-3^ g/L of LPS could directly stimulate bone loss, while a tiny concentration of LPS (10^-9^ g/L) could indirectly promote bone loss by activating the production of bone resorptive cytokines and prostaglandins.	Paula-Silva et al., 2020; Beuscher et al., 1987; Tatakis et al., 1988
	LPS could inhibit differentiation and proliferation while promoting apoptosis of osteoblasts *via* various mechanisms.	Wilson et al., 1988; Meghji et al., 1992; Tachikake-Kuramoto et al., 2014; Albus et al., 2016; Sosroseno et al., 2009
	High concentrations of *P. gingivalis* LPS could reduce mesenchymal stem cell proliferation and osteogenic differentiation, and have the capacity to inhibit activated T cells.	Tang et al., 2015
	*P. gingivalis* LPS increased the expression of RANKL *via* TLR2 in osteoblasts.	Kassem et al., 2015
	LPS of oral bacteria could stimulate Notch signaling, thus inducing IL-6 expression in macrophages. Macrophages stimulated by LPS *in vitro* showed increased expression of JAG1, implying that LPS and Notch signaling are involved in bone loss.	Wongchana and Palaga, 2012; Skokos and Nussenzweig, 2007; Tsao et al., 2011
	*P. gingivalis* LPS could modulate the expression of Wnt signaling, regulating alveolar bone health.	Nanbara et al., 2012; Maekawa et al., 2017; Tang et al., 2014
CPA	CPA from serotype c (CPA-c) of *A. actinomycetemcomitans* inhibited osteoblast cell line proliferation through a pro-apoptotic mechanism.	Yamamoto et al., 1999
Protease	Red complex pathobionts damage the epithelial tissue through the production of high protease activity which allows for the translocation of immunostimulatory molecules into tissues.	Bamford et al., 2007; Saito et al., 1997
Gingipains	Gingipains of *P. gingivalis* cleaved and degraded OPG and increased the RANKL/OPG ratio, contributing to bone loss by inducing osteoclast formation.	Tsukasaki and Takayanagi, 2019; Yasuhara et al., 2009; Akiyama et al., 2014
RagA RagB	The expression of RagA and RagB of *P. gingivalis* was increased after exposure to smoking, which could facilitate the invasion of *P. gingivalis* to the periodontium.	Bagaitkar et al., 2009
OMP29	Surface RANKL on T cells primed with *A. actinomycetemcomitans*-derived OMP29 was essential for osteoclastogenesis.	Lin et al., 2011
Td92	Td92, the surface protein of *T. denticola*, activates NLRP3 in macrophages and induces caspase-1-dependent cell death	Jun et al., 2012
	Td92 induces osteoclastogenesis *via* prostaglandin E(2)-mediated RANKL/osteoprotegerin regulation	Kim et al., 2010
Dentilisin	*T. denticola* dentilisin stimulates tissue-destructive cellular processes in a TLR2/MyD88/Sp1-dependent fashion	Ganther et al., 2021
FimA	The upregulation of FimA suppressed the host response to *P. gingivalis* by abrogating the proinflammatory response to subsequent TLR2 stimulation, and, therefore, increasing bacterial survival.	Bagaitkar et al., 2010
CDT	Stimulation of CDT of *A. actinomycetemcomitans* caused upregulation of RANKL.	Belibasakis et al., 2005
LTA	LTA of *E. faecalis* could increase the levels of NLRP3, caspase-1, and IL-1β, which resulted in bone loss.	Yin et al., 2020

LPS, lipopolysaccharide; P. gingivalis, Porphyromonas gingivalis; RANKL, receptor of nuclear factor kappa B ligand; TLR, toll-like receptor; IL, interleukin; JAG1, Jagged 1; Wnt, Wingless-integrated; CPA, capsular-like polysaccharide antigen; A. actinomycetemcomitans, Aggregatibacter actinomycetemcomitans; OPG, osteoprotegerin; Rag, Ras-related GTP-binding protein; OMP, outer membrane protein; T. denticola, Treponema denticola; NLRP3, nucleotide oligomerization domain-like receptor family pyrin domain-containing 3; FimA, fimbrilin; CDT, cytolethal distending toxin; LTA, lipoteichoic acid; E. faecalis, enterococcus faecalis; NF-κB, nuclear factor kappa B; ROS, reactive oxygen species.

### 3.1 Signaling Pathways Related to Oral Microbiota-Mediated Alveolar Bone Remodeling

#### 3.1.1 RANKL Signaling

RANKL is the master regulator of osteoclast differentiation and function. It binds to its cognate receptor on osteoclast precursors, inducing osteoclast differentiation and activation of bone resorption (Khosla, 2001). Osteoblasts, as well as osteocytes, also produce osteoprotegerin (OPG), a decoy receptor for RANKL, to block RANKL signaling, inhibiting osteoclast differentiation and bone resorption by mature osteoclasts (Khosla, 2001; Koide et al., 2013; Tsukasaki et al., 2020). An imbalance in the RANKL/OPG ratio is thought to deregulate bone remodeling, driving bone loss when the ratio exceeds that of normal physiology (Boyce and Xing, 2008).

Accumulating evidence has shown that RANKL signaling plays a critical role in alveolar bone loss in periodontitis (Belibasakis and Bostanci, 2012; Tsukasaki, 2021). Periodontal ligament cells, gingival epithelial cells, osteoblasts, osteocytes, and activated T and B cells are the major sources of RANKL in periodontal tissues (Kanzaki et al., 2002; Kawai et al., 2006; Nakashima et al., 2011; Usui et al., 2016). Patients with periodontitis have been shown to have an upregulated expression of RANKL in periodontal tissue, and the level of RANKL was highly correlated with the severity of periodontitis (Nagasawa et al., 2007); moreover, periodontitis-induced alveolar bone loss and osteoclast differentiation were markedly suppressed in RANKL-deficient mice (Tsukasaki et al., 2018). RANKL is also reportedly upregulated in periapical lesions and peri-implantitis sites (Duka et al., 2019; Kensara et al., 2021). It is also worth noting that OPG-knockout mice spontaneously developed severe alveolar bone loss, suggesting that not only the upregulation of RANKL, but also the downregulation and/or degradation of OPG is involved in periodontal bone loss (Koide et al., 2013). The RANKL/OPG ratio is associated with the degree of bone destruction in periodontitis (Bostanci et al., 2007), and an increased RANKL/OPG ratio may serve as a biomarker for the occurrence of periodontitis (Belibasakis and Bostanci, 2012; Tsukasaki, 2021).

Previous studies have indicated that RANKL could be activated directly by oral bacteria and their virulence factors (Belibasakis et al., 2005; Lin et al., 2011; Kassem et al., 2015). In osteoblasts, LPS of *P. gingivalis* increased the expression of RANKL *via* toll-like receptor 2 (TLR2) (Kassem et al., 2015). Td92 of *T. denticola* induced RANKL expression and promoted osteoclast formation *via* prostaglandin E(2)-dependent mechanism (Kim et al., 2010). Stimulation of gingival fibroblasts and periodontal ligament cells with cytolethal distending toxin from *A. actinomycetemcomitans* caused upregulation of RANKL (Belibasakis et al., 2005). Additionally, surface RANKL on T cells primed with *A. actinomycetemcomitans*-derived outer membrane protein 29 was essential for osteoclastogenesis (Lin et al., 2011). RANKL could also be regulated indirectly by the oral microbiota *via* an induced immune response. To summarize, the oral microbiota and its metabolites induce the production of inflammatory cytokines (e.g., IL-1, IL-6, and TNF-α), macrophage inflammatory protein-1, and macrophage chemoattractant protein by different immune cells, including neutrophils, monocytes, macrophages, dendritic cells, T cells, and B cells, leading to the increased expression of RANKL (Brunetti et al., 2005; Rogers et al., 2007; Hung et al., 2014; Tompkins, 2016). An animal study demonstrated that activation of T cells by oral bacteria caused RANKL-induced bone loss (Mahamed et al., 2005). Moreover, certain proteases derived from oral bacteria (e.g., gingipains of *P. gingivalis*) cleave and degrade OPG, thereby increasing the RANKL/OPG ratio and contributing to bone loss by inducing osteoclast formation (Yasuhara et al., 2009; Akiyama et al., 2014; Tsukasaki and Takayanagi, 2019). It is worth noting that osteoclast formation can also be induced by inflammatory chemokines and cytokines independent of RANKL (Hotokezaka et al., 2007; Valerio et al., 2015).

#### 3.1.2 Notch Signaling

The Notch signaling pathway is considered a double-edged sword in osteoclastogenesis depending on the status of the osteoclasts and the expression of certain receptors and ligands (Pakvasa et al., 2020). However, in the context of oral microbiota-mediated alveolar bone remodeling, Notch signaling is mainly involved in alveolar bone resorption. A series of studies demonstrated that the Notch signaling pathway is in a complex relationship with proinflammatory cytokines and bone resorption regulators. Alveolar bone resorption in periodontitis and apical periodontitis is mediated through an increase in Notch receptors on the immune cell surface and stimulation of Notch-receptor intracellular domain translocation into the nucleus (Jakovljevic et al., 2019; Djinic Krasavcevic et al., 2021). Furthermore, LPS of oral bacteria can stimulate Notch signaling, thus inducing IL-6 expression in macrophages (Wongchana and Palaga, 2012). Jagged 1 is a cell surface ligand that interacts with receptors in the Notch signaling pathway. Macrophages stimulated by LPS *in vitro* exhibited increased expression of Jagged 1 (Skokos and Nussenzweig, 2007; Tsao et al., 2011). These studies provided evidence that LPS in conjunction with Notch signaling can activate cells that are involved in osteoimmunology-mediated bone loss. It will be of interest to study lineage-specific genes in the Notch-signaling-pathway knockout model to identify the role of this pathway in alveolar bone loss mediated by the oral microbiota.

#### 3.1.3 Wnt Signaling

Mounting evidence indicates that Wnt signaling is essential for the control of bone mass by regulating the activity of both osteoblasts and osteoclasts. As noted above, the ratio of RANKL/OPG is key for bone resorption. Interestingly, the Wnt pathway can increase the production of OPG, decreasing the RANKL/OPG ratio and blocking RANKL-induced osteoclastogenesis (Zhong et al., 2014). The Wnt signaling pathway is involved in periodontitis, apical periodontitis, and peri-implantitis (Napimoga et al., 2014). Wnt5a is an activating ligand of non-canonical Wnt signaling pathways and plays important roles in the inflammatory response and bone development/remodeling (Zhong et al., 2014). It has been shown to enhance RANK expression in osteoclast precursors by engaging receptor tyrosine kinase-like orphan receptor 2 to activate Jun N-terminal kinase and recruiting c-Jun to the RANK gene promoter, thereby enhancing RANKL-induced osteoclastogenesis (Maeda et al., 2012). In a clinical study, the mRNA expression of Wnt5a was higher in gingival tissues from individuals with periodontitis and peri-implantitis compared to that from healthy controls (Nanbara et al., 2012; Zhang et al., 2020). Further evidence has been derived from *in vitro* and animal studies. Wnt5a was upregulated in macrophages and monocytic cell line THP-1 following stimulation with *P. gingivalis* and LPS, respectively (Nanbara et al., 2012; Maekawa et al., 2017; Zhang et al., 2020). In macrophages, the induction of Wnt5a was dependent on lectin-type oxidized low density lipoprotein receptor-1 and TLR4. Wnt5a knockdown significantly impaired the production of IL-1β, macrophage chemoattractant protein 1, and matrix metalloproteinase-2 upon induction by *P. gingivalis* (Zhang et al., 2020). In THP-1 cells, this process is dependent upon NF-κB signaling (Nanbara et al., 2012; Maekawa et al., 2017). In a study using a rat model of apical periodontitis, inhibition of the Wnt/β-catenin signaling by Dickkopf-1 attenuated alveolar bone loss *via* regulation of bone coupling *in vivo* (Tan et al., 2018). Conversely, in rat bone marrow mesenchymal cells, Wnt/β-catenin signaling was inhibited by LPS of *P. gingivalis* and the cells exhibited decreased osteogenic potential (Tang et al., 2014). Thus, more research is required, especially in the form of *in vivo* studies, to clarify the role of Wnt signaling and related pathways in alveolar bone loss.

#### 3.1.4 NLRP3 Inflammasome

The NLRP3 inflammasome is an essential component of the natural immune system (Lamkanfi and Dixit, 2014) and a critical mediator of alveolar bone loss. The reported intensity of NLRP3 expression was statistically significantly higher in tissues from patients with periodontitis than that from healthy controls (Huang et al., 2015; Xue et al., 2015). In experimental mice models, alveolar bone loss was correlated with caspase-1 activation by macrophages and elevated concentrations of IL-1β, which is mainly regulated by the NLRP3 inflammasome (Zang et al., 2020; Chen et al., 2021). NLRP3 knockout mice exhibited a higher bone mass and reduced osteoclast precursors and differentiation compared with wild-type mice. More importantly, an NLRP3 inflammasome inhibitor statistically significantly improved alveolar bone mass with reduced proinflammatory cytokine production and increased osteogenic gene expression in mice with periodontitis (Zang et al., 2020; Chen et al., 2021).

Many studies have been conducted to determine whether the NLRP3 inflammasome can be regulated by the oral microbiota. The NLRP3 inflammasome can recognize oral pathogen-associated molecular patterns and host-derived danger-signaling molecules, and activate the pro-inflammatory protease, caspase-1 (Yu et al., 2021). These pathogen-associated molecular patterns include LPS, peptidoglycan, and viral double-stranded RNA (Brown et al., 2011; Amari and Niehl, 2020). After activation, caspase-1 cleaves the precursors of IL-1β and IL-18 to produce mature cytokines (Menu and Vince, 2011). IL-1β further induces secretion of RANKL and activates osteoclasts, which can cause a series of inflammatory responses (Nakashima et al., 2000). Activated caspase-1 specifically recognizes and cleaves gasdermin D to mediate cell pyroptosis (Wang K. et al., 2020). Pathogens of periapical periodontitis and periodontitis can activate NLRP3 *in vitro*. For instance, lipoteichoic acid from *Enterococcus faecalis*, the most common pathogen in periapical periodontitis, can induce the expression of NLRP3 and increase the levels of caspase-1 and IL-1β, thus resulting in bone loss (Yin et al., 2020). It is worth mentioning that inhibition of the NLRP3 inflammasome can effectively alleviate those effects (Yin et al., 2020). Td92, the surface protein of *T. denticola*, activates NLRP3 in macrophages and induces caspase-1-dependent cell death (Jun et al., 2012). *A. actinomycetemcomitans* can also activate NLRP3 (Zhao et al., 2014). In one study, heat-killed *A. actinomycetemcomitans* injected into the gum tissues of caspase-1-knockout mice statistically significantly decreased alveolar bone resorption in comparison with wild-type mice (Rocha et al., 2020). Furthermore, knockdown of NLRP3 using small interfering RNA in *A. actinomycetemcomitans*-infected osteoblasts attenuated apoptosis, which suggests that *A. actinomycetemcomitans* invasion of the alveolar bone surface may directly promote osteoblast apoptosis through the NLRP3 inflammasome (Zhao et al., 2014). There is also indirect evidence that differentiation of THP-1 cells into macrophage-like cells, induced by *P. gingivalis* and *F. nucleatum*, is NLRP3- and caspase-1-dependent (Kawahara et al., 2020). In MC3T3-E1 cells, stimulation with *P. gingivalis* resulted in the protein kinase R-mediated increase in NLRP3 expression *via* activation of NF-κB (Yoshida et al., 2017).

#### 3.1.5 Gingival Solitary Chemosensory Cells

Solitary chemosensory cells (SCCs) are epithelial sentinels that utilize bitter taste receptors and coupled taste signaling elements to detect pathogen metabolites, stimulating host defenses to control the infection (O'Leary et al., 2019). Previously, our research team discovered that SCCs were present in mouse gingival junctional epithelium where they expressed several bitter taste receptors and the taste signaling elements, α-gustducin, transient receptor potential cation channel subfamily M member 5, and phospholipase C β2 (Zheng et al., 2019). The commensal oral microbiome was altered and natural alveolar bone loss was accelerated in α-gustducin knockout mice. In a model of ligature-induced periodontitis, knockout of taste signaling molecules or the genetic absence of gingival SCCs increased the bacterial load, reduced bacterial diversity, and caused a pathogenic shift in the microbiota, leading to greater alveolar bone loss. Topical treatment with bitter denatonium to activate gingival SCCs upregulated the expression of antimicrobial peptides and ameliorated ligature-induced periodontitis in wild-type but not in α-gustducin^−/−^ mice (Zheng et al., 2019). These results demonstrated that gingival SCCs may provide a promising target for treating periodontitis by harnessing the innate immunity to regulate the oral microbiome.

### 3.2 Osteoimmunity in Alveolar Bone Loss Mediated by Oral Microbiota

Osteoimmunology has developed because of the close interplay between the immune system and bone metabolism (Rho et al., 2004). Mediation of the immune response by the oral microbiota, especially pathogens, is critical for bone homeostasis. Dysbiosis in the oral microbial community influences the host immune response, and the immunoinflammatory reaction may shape the composition of the oral microbiota and contribute to the homeostatic relationship between microbiota and host (Hajishengallis, 2014). Oral microbiota-triggered innate and acquired immune responses are considered to be a double-edged sword in alveolar bone loss. The complement system, phagocytosis, the inducible nitric oxide synthase-mediated immune responses, and the production of antigen-specific immunoglobulins protect hosts from harmful bacteria (Jiao et al., 2014). For example, mice lacking inducible nitric oxide synthase, P-selectin, or intercellular adhesion molecule 1 are susceptible to alveolar bone loss after *P. gingivalis* infection (Baker et al., 2000; Fukada et al., 2008). However, an imbalance in the homeostasis between bacteria and host immune responses culminates in bone resorption. Bacteria possess a variety of immunostimulatory molecules, some of which induce recruitment of immune cells and others secretion of TNF-α and IL-1β from immune cells (Takeuchi and Akira, 2010). Red complex pathobionts (*P. gingivalis*, *T. denticola*, and *Tannerella forsythia*) damage the epithelial tissue by stimulating proteases that allow the translocation of immunostimulatory molecules into tissues (Saito et al., 1997; Bamford et al., 2007). In response to oral bacteria, IL-6, TNF-α, and IL-1β are secreted from neutrophils and macrophages that are recruited to damaged gingival tissues (Takeuchi and Akira, 2010). Nucleotide oligomerization domain-like receptor 1 ligands produced by certain bacteria possess the ability to recruit neutrophils that secrete inflammatory cytokines (e.g., TNF and IL-1) to alter the RANKL/OPG ratio in activated T cells, B cells, and osteoblasts, causing alveolar bone loss at damaged gingival sites (Hasegawa et al., 2006; Masumoto et al., 2006). Pathogenic T_H_17 cells stimulated by bacterial invasion evoke a mucosal immune response for protection against pathogens while inducing bone damage (Tsukasaki et al., 2018). Based on the accumulated evidence, we speculate that moderate immune responses induced by oral microbiota may be beneficial for alveolar bone, whereas the expression of large numbers of pro-inflammatory cytokines induced by excessive immune responses promote alveolar bone loss.

In the oral cavity, the oral microbiome, host immune system, and alveolar bone co-exist and interact. Osteomicrobiology bridges the gap between the microbiome and osteoimmunology. Osteomicrobiology and osteoimmunology are inseparable but have distinguishing characteristics. The challenge is to maintain homeostasis in the oral microbiome, moderate inflammation, and remodeling of the alveolar bone.

## 4 Factors That Affect Oral Microbiota-Mediated Alveolar Bone Metabolism

The oral microbiota is directly or indirectly responsible for most alveolar bone loss, however, the relationship is modified by various interacting factors, including smoking, blood glucose level, estrogen concentration and probiotics. Studies in this field have provided details of the crosstalk between these factors. This section aims to offer an overview of how these factors influence oral microbiota-mediated alveolar bone metabolism.

Life events and general health conditions can affect the bone metabolism (Feres et al., 2016). For instance, obesity and hypertension have an impact on the oral microbial composition and regulate alveolar bone metabolism (Del Pinto et al., 2020; Khan et al., 2020). Smoking, diabetes mellitus (DM), and estrogen deficiency are associated with systemic bone loss, including osteoporosis and alveolar bone resorption (Weitzmann and Pacifici, 2006; Straka et al., 2015; Wang X. et al., 2020). Clinical and experimental studies have revealed a higher prevalence of periodontitis, periapical periodontitis, or peri-implantitis associated alveolar bone resorption in patients/animal models who smoke or with DM/estrogen deficiency (Duarte et al., 2004; Lima et al., 2013; Penoni et al., 2017; Gupta et al., 2020; Ford and Rich, 2021). Data from cross-sectional studies have also demonstrated that the severity of periodontitis and alveolar bone loss were positively correlated with the amount of daily smoking (Hujoel et al., 2003). The relationship of DM and periodontal disease is bidirectional, compromised management of either one would negatively affect the other one (Radaic and Kapila, 2021). Positive management of these factors exhibited beneficial effect on alveolar bone remodeling. For example, estrogen therapy is an effective method for improving alveolar bone density in post-menopausal patients with osteoporosis (Ronderos et al., 2000; Bhavsar et al., 2016).

Probiotics have been used to induce beneficial skeletal effects for it can alter the composition and/or the metabolic activity of the gut microbiota, and regulate the immune response in the host, thereby providing beneficial effects for bone health (Abboud and Papandreou, 2019; Pan et al., 2019; Schepper et al., 2020). Randomized clinical studies and animal studies demonstrated that oral administration of certain probiotics is a useful strategy for the management of periodontitis, periapical periodontitis, and peri-implantitis (Huck et al., 2020; Cosme-Silva et al., 2021; Kumar et al., 2021). Increasing evidence have shown that these factors impact the alveolar bone metabolism mainly through modulating the oral microbiota and the host immune response (osteomicrobiological modulatory effects).

### 4.1 Alter the Composition and Virulence of the Oral Microbiota

The factors can alter the composition and virulence factors of oral microbiota, thus affecting alveolar bone metabolism directly or indirectly. In smoking-related periodontitis or peri-implantitis, the microbial profile is distinct from that in non-smokers, and there are statistically significant differences in the prevalence and enrichment of disease-associated and health-compatible microorganisms (Shchipkova et al., 2010; Duan et al., 2017; Stokman et al., 2017; Naseri et al., 2020). Levels of disease-associated pathogens have been revealed to decrease following smoking cessation (Delima et al., 2010). The expression of several virulence factors of *P. gingivalis* (e.g., fimbrilin and Ras-related GTP-binding proteins A and B) increased after exposure to smoking, which could suppress the host response by abrogating the proinflammatory response to subsequent TLR2 stimulation, and therefore could facilitate the invasion of *P. gingivalis* into the periodontium (Bagaitkar et al., 2009). Furthermore, the expression of capsular polysaccharide is inhibited by smoking, thus promoting the colonization of *P. gingivalis* and enhancing both inter- and cross-species interaction of *P. gingivalis*, aggravating the alveolar bone loss (Zhang et al., 2019).

Hyperglycemia is able to cause dysbiosis of the oral microbiota, with a statistically significant enrichment of *Leptotrichia, Staphylococcus, Catonella*, and *Bulleidia* genera, contributing to aggravation of alveolar bone loss (Wang et al., 2019). Hintao et al. demonstrated that *T. denticola, Streptococcus sanguinis, Prevotella nigrescens, Staphylococcus intermedius*, and *Streptococcus oralis* were statistically significantly enriched in the supragingival plaque of individuals with type 2 DM compared with individuals without DM (Hintao et al., 2007). DM can also increase the pathogenicity of the dysbiotic oral microbiota. A study demonstrated that DM enhanced IL-17 expression and altered the oral microbiome to increase its pathogenicity (Xiao et al., 2017). Compared with the oral microbiomes of healthy mice, the pathogenic oral microbiomes of diabetic mice statistically significantly exacerbated periodontal inflammation and bone loss when transferred to GF mice (Xiao et al., 2017).

Postmenopausal women with endogenous estrogen deficiency exhibited a progressive loss in radiographic alveolar crestal height over 5 years, and that loss was associated with a change in the subgingival microbiome (LaMonte et al., 2021). The abundance of *P. gingivalis* and *T. forsythensis* were increased and were revealed to be critical in the etiology of periodontitis in postmenopausal women (Brennan et al., 2007). Cohort studies demonstrated that estrogen therapy improved periodontal probing depth and tooth mobility, with decreased levels of *P. gingivalis*, *P. intermedia*, and *T. forsythia* being detected in subgingival plaque (López-Marcos et al., 2005; Tarkkila et al., 2010). Changes in estrogen levels may cause the gums to become more susceptible to plaque, leading to a much higher risk of advanced periodontitis (Suresh and Radfar, 2004). Furthermore, estrogen-deficient conditions interfere with the oral microbiota by increasing the levels of certain bacteria in saliva and influencing the progression of periapical bone loss (Lucisano et al., 2021).

In contrast to causing oral microbiota dysbiotic, probiotics facilitate the change of abundance towards health-favoring commensals, modulating the oral microecology. Animal studies revealed that topical application of *Lactobacillus brevis* cluster of differentiation (CD) 2 attenuated alveolar bone loss, with a reduction in anaerobic bacteria and an increase in aerobic bacteria in mice (Maekawa and Hajishengallis, 2014). Oliveira et al. discovered that topical application of *Bifidobacterium lactis* HN019 reduced bone destruction, decreased the proportions of *Veillonella parvula*, *Capnocytophaga sputigena*, *E. corrodens*, and *P. intermedia*-like species, and increased the proportions of *Actinomyces* and *Streptococcus*-like species (Oliveira et al., 2017). *In vitro* studies have demonstrated that certain probiotics exhibit inhibitory activity against endodontic pathogens (Bohora and Kokate, 2017a; Bohora and Kokate, 2017b). Probiotic *Akkermansia muciniphila* was revealed to reduce gingipain transcription by *P. gingivalis*, thereby decreasing inflammatory cell infiltration and alleviating alveolar bone loss (Huck et al., 2020). Recently, we discovered that administration of probiotics enriched butyrate-producing genera of gut microbiota, improved intestinal barrier function, and decreased gut permeability, thus preventing inflammatory alveolar bone resorption in ovariectomized rats (Jia et al., 2021).

### 4.2 Modulate the Host Immune Response

The factors could also influence the interaction between oral microbiota and alveolar bone *via* modulating the innate and adaptive host immune response. Smoking impairs chemotaxis and phagocytosis of neutrophils in the periodontal tissues and inhibits serum immunoglobulin G antibodies against periodontal pathogens, exerting a “protective” effect on pathogens (Guntsch et al., 2006; Vlachojannis et al., 2010). Furthermore, smoking indirectly modulates the oral microbiota and host immune response by inducing the generation of reactive oxygen species (ROS), which have been found to be essential for osteoclastogenesis (Matthews et al., 2011). DM altered the equilibrium of osteoclasts and osteoblasts in the alveolar bone by shaping the oral microbial balance, and by increasing the concentrations of inflammatory mediators (e.g., TNF), the RANKL/OPG ratio, advanced glycation end products, and ROS (Wu et al., 2015; Graves et al., 2019). Hyperglycemia inhibits osteoblastic differentiation as well as new bone formation, exacerbates alveolar bone resorption, and enhances peri-implant inflammation, frequently causing implant failure (Chrcanovic et al., 2014). Estrogen deficiency can also inhibit the production of cytokines triggered by dysbiotic microbiota, lower the RANKL/OPG ratio, and stimulate the production of transforming growth factor β by osteoblasts, resulting in a decrease in osteoclast quantity and activity (Hughes et al., 1996; Riggs, 2000). Postmenopausal estrogen deficiency induces the production of TNF-α and RANKL in T cells, and influences the activities of bone multicellular units, resulting in a reduction in the ratio of bone deposition by osteoblasts to bone resorption by osteoclasts, enhancing the progression of alveolar bone loss in patients with periodontitis or apical periodontitis (Lerner, 2006; D'Amelio et al., 2008). The above studies provide evidence that, smoking, DM, and estrogen deficiency exacerbate the loss of alveolar bone by promoting the invasion of pathogenic bacteria and aggravating the inflammatory response.

Contrarily, probiotics have a protective effect against alveolar bone loss by modifying immunoinflammatory parameters. *L. brevis* CD2 treatment resulted in statistically significantly less bone loss and a downregulation of TNF, IL-1β, IL-6, and IL-17A compared to placebo treatment (Maekawa and Hajishengallis, 2014). The group treated with *B. lactis* HN019 exhibited increased expressions of OPG and β-defensins, while decreased expressions of IL-1β and RANKL compared to the control group (Oliveira et al., 2017). Pazzini et al. also revealed that oral supplementation with probiotic *Bacillus subtilis* was beneficial for bone remodeling by reducing the number of osteoclasts adjacent to the tooth root during orthodontic movement in mice (Pazzini et al., 2017).

Collectively, the composition of the oral microbiota and host immune response varies depending on dietary diversification, medicine used, hormonal changes, general health conditions, and age (Feres et al., 2016). Many factors could influence the osteomicrobiological modulatory effect in physiological or pathological conditions. The factors mentioned above interact with each other in antagonistic and synergistic ways to influence oral microbiota-mediated alveolar bone health. For example, estrogen depletion and streptozotocin-induced DM promoted more pronounced periodontal tissue deterioration than each did in isolation (Sasso et al., 2020). Probiotic administration has a protective effect on the mandibular bone mineral density in rats exposed to cigarette smoke inhalation (Levi et al., 2019). More studies are needed to determine the mechanisms by which these factors impact oral microbiota-mediated alveolar bone metabolism. These studies would facilitate the discovery of critical targets and the development of strategies for manipulating the microbiota to induce beneficial skeletal effects.

## 5 Critical Techniques for Oral Osteomicrobiology Research

The oral cavity harbors over 700 species, including bacteria, fungi, viruses, archaea, and protozoans, although only approximately 70% of them can be cultivated, based on the expanded Human Oral Microbiome Database (Verma et al., 2018). With the advances in rapid, low-cost sequencing technologies and next-generation sequencing-based platforms, it is possible to quantitatively characterize the composition and putative functions of microbial communities (Human Microbiome Project Consortium, 2012). 16S ribosomal DNA sequencing has greatly contributed to revealing the composition of the oral microbiome. It allows identification of bacteria at a highly accurate genus level by amplifying one or more high-variation zones, such as V1, V2, V3, and V4 regions. However, this method does not provide the full-length DNA sequence; thus, it cannot be used to distinguish species and strains, nor to identify fungi and viruses (Janda and Abbott, 2007). To overcome this drawback, whole genome sequencing, metatranscriptomics, metaproteomics, and metabolomics can be used to identify strains present in the oral microbiome, and to detect microbial genes, proteins, and metabolites that have an impact on diseases (Human Microbiome Project Consortium, 2012). Although analysis of next-generation sequencing-derived sequences remains challenging, it has greatly improved our understanding of the relationships between the oral microbiota and alveolar bone health. The importance of the microbiota has been confirmed and new insights have been gained on their effects on bone physiology (Ohlsson and Sjögren, 2018).

Animal models are also useful for studying the role of the oral microbiota in alveolar bone mass regulation. Two prominent models, GF mice and humanized mice, are of great importance for *in vivo* studies of host microbial interaction. GF mice have been employed to explore the role of oral pathobionts in dysbiosis and bone loss during periodontitis for more than half a century (Baer and Newton, 1960). The model can be used to investigate the effects of both mono-infection and polymicrobial colonization on alveolar bone. Importantly, the molecular mechanism by which the oral microbiota affects bone mass can also be demonstrated using genetically engineered GF mouse models in which selected genes are deleted or overexpressed. The most typical example is monospecies inoculation (of e.g., *P. gingivalis*) at the ligature site to evaluate the effects of infection on alveolar bone loss (Graves et al., 2008). Recently, to better reflect real world conditions, researchers introduced a polymicrobial synergy and dysbiosis model to evaluate the features of periodontal inflammation and alveolar bone loss. That model disclosed that dysbiosis of the periodontal microbiota signifies an imbalance in the relative abundance or influence of microbial species within the ecosystem compared to physiological conditions, leading to sufficient alterations in the host–microbial crosstalk to mediate destructive inflammation and bone loss (Hajishengallis and Lamont, 2012; Bowen et al., 2018). Gao et al. used *P. gingivalis*, *T. denticola*, *T. forsythia*, and *F. nucleatum* as polymicrobial oral inoculum in BALB/cByJ mice, demonstrating that it triggered statistically significant alveolar bone loss, a heightened antibody response, an elevated cytokine immune response, and a statistically significant shift in viral diversity and virome composition (Gao et al., 2020). In addition, mouse models infected with a combination of *P. gingivalis*, *A. actinomycetemcomitans*, *T. denticola, T. forsythia*, and *F. nucleatum* (Graves et al., 2008), or *Streptococcus gordonii*, *V. parvula*, and *F. nucleatum* (Marchesan et al., 2018), as well as other bacterial combinations (Polak et al., 2009; Settem et al., 2012; Tan et al., 2014) were developed to investigate the role of oral bacteria in alveolar bone loss *in vivo*. However, these models are imperfect imitations of the human microbial systems. Therefore, the establishment of a humanized gnotobiotic mouse model by transplantation of the oral microbiota into GF mice is necessary and will be a powerful tool for future studies.

Additionally, to study the ecology and functionality of microbial communities in a controlled yet accurate way, synthetic microbial communities have received increasing attention. Synthetic microbial communities are an emerging research field at the intersection of synthetic biology and microbiomes (Estrela et al., 2021). A synthetic microbial community is created by co-culturing two or more microbial populations under well-defined conditions. It can also include genetically engineered organisms. Synthetic microbial communities that retain the key features of their natural counterparts can act as a model system to study the ecology and function of microbial communities with the advantages of low complexity, high controllability, and good stability (Estrela et al., 2021). This approach was originally developed to provide functional and mechanistic insights into plant-plant microbiome interactions (Liu et al., 2019). Now, it is widely used in biological treatment, focusing on fuel production, high value-added chemical synthesis, and pollutant degradation (Liu et al., 2019). Niu et al. obtained a greatly simplified synthetic bacterial community consisting of seven strains representing the most dominant phyla found in maize roots. By using a selective culture-dependent method to track the abundance of each strain, they discovered that the removal of only *Enterobacter cloacae* led to the complete loss of the community, with *Curtobacterium pusillum* taking over, suggesting that *E. cloacae* is the keystone species in their model ecosystem (Niu et al., 2017). Synthetic microbial ecologies were also proposed as simple and controllable model systems to facilitate bacteria-driven phthalic acid ester biodegradation, providing novel insights for developing effective bioremediation solutions (Hu et al., 2021).

Synthetic microbial communities, combined with systems biology (Estrela et al., 2021) and other experimental technologies, allow the prediction of the ecological stability of the communities and their key species, and thus may further advance the understanding of oral microbiota-alveolar bone relationships. Based on related studies in other fields (Liu et al., 2019), we propose the following workflow for synthetic microbial communities in osteomicrobiology: (1) sample collection: collecting dental plaque or saliva; (2) isolation: isolating single species by colony picking, limiting dilution, and cell sorting; (3) identification: identifying the culture using barcoded sequencing and Sanger sequencing; (4) culture collection: preserving bacteria using glycerol solution, and analyzing the proportion and relative abundance of available strains by comparing the bacterial reservoir constructed using natural samples; (5) correlation analysis: selecting the experimental strains according to the correlation between operational taxonomic unit abundance and phenotype, network analysis, and taxonomy; (6) functional analysis: inoculating single or multiple species into GF mice, and observing the changes in the phenotype and structure of the oral microbial community. It is worth noting that the composition of the microbial communities is critical for the services and functions they provide, and learning how to manipulate such is of great importance. Therefore, the following requirements should be considered when selecting the microbial communities: there must be variation between competing communities in terms of community traits, communities must be able to replicate, and the community trait must be heritable (Buss, 1983). Furthermore, it has recently become possible to automate synthetic microbiome design (Tran and Prindle, 2021). For example, computer-guided design has been used to select optimal microbial consortia that promote the activation of regulatory T cells in a gut microbiota-immune system model (Stein et al., 2018).

## 6 Conclusion

Collectively, the evidence indicates a close connection between the oral microbiota and bone health. The oral microbiota plays important roles in post-natal jawbone development, physiological alveolar bone loss, and, particularly, pathological alveolar bone loss associated with oral diseases such as periodontitis, apical periodontitis, and peri-implantitis. Under pathological conditions, oral pathogenic microbes and microbial dysbiosis induce catabolic disruption of osteoclast-osteoblast-mediated bone remodeling, which leads to alveolar bone loss. RANKL, Notch, and Wnt signaling, as well as the NLRP3 inflammasome are major pathways involved in this process, and osteoimmunity is the key bridge between microbiota and bone. More studies are needed to identify which oral microbes contribute to alveolar bone loss and determine the underlying mechanisms by which oral microbial dysbiosis is related to alveolar bone metabolism. Synthetic microbial communities, combined with a multi-omics approach and mouse models are anticipated to provide new insights into the oral microbiota-alveolar bone relationship. In addition, many factors, such as probiotics, smoking, DM, and the estrogen concentration interact antagonistically and synergistically in influencing oral microbiota-mediated alveolar bone health. With the advances in experimental and clinical studies and the growth of personalized medicine, perhaps, in the future, such factors may be manipulated to alter the composition of the oral microbiome and effectively prevent alveolar bone loss.

Here, we propose use of the term “oral osteomicrobiology” for the rapidly emerging research field of the role of oral microbes in alveolar bone health, bridging the gaps between oral microbiology, immunology, and alveolar bone physiology or alveolar bone pathology. Oral osteomicrobiology refers to investigations on the role of the oral microbiota in alveolar bone health and disease; the mechanisms by which they regulate post-natal jawbone development as well as physiological and pathological alveolar bone loss; and the experimental methods and technologies developed for associated research.

## Author Contributions

XC drafted the manuscript. XZ, CL, and XX edited and added valuable insights to the manuscript. All authors contributed to the article and approved the submitted version.

## Funding

This study was supported by the National Natural Science Foundation (81771099 to XX, 81870754 to XZ); the Sichuan University Postdoctoral Interdisciplinary Innovation Fund to XC; the Research and Develop Program, West China Hospital of Stomatology Sichuan University to XC (RD-02-201908); and the Research Funding from West China Hospital of Stomatology Sichuan University to XC (RCDWJS2021-16).

## Conflict of Interest

The authors declare that the research was conducted in the absence of any commercial or financial relationships that could be construed as a potential conflict of interest.

## Publisher’s Note

All claims expressed in this article are solely those of the authors and do not necessarily represent those of their affiliated organizations, or those of the publisher, the editors and the reviewers. Any product that may be evaluated in this article, or claim that may be made by its manufacturer, is not guaranteed or endorsed by the publisher.

## Abbreviations

CD, cluster of differentiation; DM, diabetes mellitus; GF, germ-free; IL, interleukin; LPS, lipopolysaccharide; NF-κB, nuclear factor kappa B; NLRP3, nucleotide oligomerization domain-like receptor family pyrin domain-containing 3; OPG, osteoprotegerin; RANKL, receptor activator of nuclear factor kappa B ligand; SCC, solitary chemosensory cell; SPF, specific pathogen-free; TNF-α, tumor necrosis factor-α; Wnt, Wingless-integrated.
